# Comprehensive Analysis of 5-Aminolevulinic Acid Dehydrogenase (*ALAD*) Variants and Renal Cell Carcinoma Risk among Individuals Exposed to Lead

**DOI:** 10.1371/journal.pone.0020432

**Published:** 2011-07-20

**Authors:** Dana M. van Bemmel, Paolo Boffetta, Linda M. Liao, Sonja I. Berndt, Idan Menashe, Meredith Yeager, Stephen Chanock, Sara Karami, David Zaridze, Vsevolod Matteev, Vladimir Janout, Hellena Kollarova, Vladimir Bencko, Marie Navratilova, Neonilia Szeszenia-Dabrowska, Dana Mates, Alena Slamova, Nathaniel Rothman, Summer S. Han, Philip S. Rosenberg, Paul Brennan, Wong-Ho Chow, Lee E. Moore

**Affiliations:** 1 Cancer Prevention Fellowship Program, Office of the Director, National Cancer Institute, Bethesda, Maryland, United States of America; 2 Division of Cancer Epidemiology and Genetics, National Cancer Institute, Bethesda, Maryland, United States of America; 3 Core Genotyping Facility, National Cancer Institute, Gaithersburg, Maryland, United States of America; 4 The Tisch Cancer Institute, Mount Sinai School of Medicine, New York, New York, United States of America; 5 International Prevention Research Institute, Lyon, France; 6 Institute of Carcinogenesis, Cancer Research Centre, Moscow, Russia; 7 Department of Preventive Medicine, Faculty of Medicine, Palacky University, Olomouc, Czech Republic; 8 Institute of Hygiene and Epidemiology, 1st Faculty of Medicine, Charles University, Prague, Czech Republic; 9 Masaryk Memorial Cancer Institute, Brno, Czech Republic; 10 Institute of Occupational Medicine, Lodz, Poland; 11 Institute of Public Health, Bucharest, Romania; Brigham & Women's Hospital, and Harvard Medical School, United States of America

## Abstract

**Background:**

Epidemiologic studies are reporting associations between lead exposure and human cancers. A polymorphism in the 5-aminolevulinic acid dehydratase (*ALAD*) gene affects lead toxicokinetics and may modify the adverse effects of lead.

**Methods:**

The objective of this study was to evaluate single-nucleotide polymorphisms (SNPs) tagging the *ALAD* region among renal cancer cases and controls to determine whether genetic variation alters the relationship between lead and renal cancer. Occupational exposure to lead and risk of cancer was examined in a case-control study of renal cell carcinoma (RCC). Comprehensive analysis of variation across the *ALAD* gene was assessed using a tagging SNP approach among 987 cases and 1298 controls. Occupational lead exposure was estimated using questionnaire-based exposure assessment and expert review. Odds ratios (OR) and 95% confidence intervals (CI) were calculated using logistic regression.

**Results:**

The adjusted risk associated with the *ALAD* variant rs8177796^CT/TT^ was increased (OR = 1.35, 95%CI = 1.05–1.73, p-value = 0.02) when compared to the major allele, regardless of lead exposure. Joint effects of lead and *ALAD* rs2761016 suggest an increased RCC risk for the homozygous wild-type and heterozygous alleles (^GG^OR = 2.68, 95%CI = 1.17–6.12, p = 0.01; ^GA^OR = 1.79, 95%CI = 1.06–3.04 with an interaction approaching significance (p_int_ = 0.06).. No significant modification in RCC risk was observed for the functional variant rs1800435^(K68N)^. Haplotype analysis identified a region associated with risk supporting tagging SNP results.

**Conclusion:**

A common genetic variation in *ALAD* may alter the risk of RCC overall, and among individuals occupationally exposed to lead. Further work in larger exposed populations is warranted to determine if *ALAD* modifies RCC risk associated with lead exposure.

## Introduction

Lead is a naturally occurring heavy metal used in the manufacturing of consumer products including; batteries, paints, metal products (such as sheet metal), cable covering, and ceramic glaze. The wide-spread use of lead in manufacturing results in a continued occupational exposure to lead world-wide. The toxic effects of acute lead exposure are well-established. At high levels, lead exposure results in adverse effects on hematopoietic, gastrointestinal, urinary, cardiovascular, and nervous systems [1]. At lower doses, chronic lead exposure has been associated with aberrant cognitive development in children, anemia, hypertension, and the development of neurological disorders [2], [3], [4], [5]. The International Agency for Research on Cancer (IARC) classifies inorganic lead as a probable human carcinogen (Group 2A), based on sufficient evidence from animal studies and limited epidemiologic research [6] .

One of the most important mechanisms of lead toxicity is its inhibition of key enzymes within the heme biosynthetic pathway. The most well characterized interaction is between lead and the second enzyme in the heme biosynthetic pathway known as 5-aminolevulinic acid dehydratase (ALAD). The gene that encodes ALAD exists in two polymorphic forms (*ALAD_1_* and *ALAD_2_ [SNP rs1800435]*) that may influence an individual's susceptibility to lead poisoning [7]. The difference between the two forms of ALAD is an amino acid substitution of an asparagine for lysine residue 59; resulting from a single amino acid change in position 177 of the coding region [8]. This substitution results in an increased affinity of ALAD_2_ for lead compared to ALAD_1_ [9]. Rodents genetically modified to have an extra copy of the *ALAD* gene accumulated an average of 2.4-fold higher levels of lead in the kidney, 4.1-times higher in the liver, and 2.5-fold higher in the brain compared to those with a single copy exposed to the same doses [10].

Recently, we reported an increase in renal cell carcinoma (RCC) risk among participants in the Central Eastern European Renal Cell Cancer Study occupationally exposed to lead [11]. Due to the important role of ALAD in lead metabolism, we hypothesized that common genetic variation in *ALAD* may alter lead exposure and be associated with RCC risk. To test this hypothesis, we comprehensively evaluated 19 single nucleotide polymorphisms (SNPs) in and around the *ALAD* gene in this study.

## Materials and Methods

### Study Population

The Central and Eastern European Renal Cell Cancer Study (CEERCC) is a hospital based case-control study conducted in seven centers across 4 Eastern European countries (Moscow, Russia; Bucharest, Romania; Lodz, Poland; and Prague, Olomouc, Ceske Budejovice and Brno, Czech Republic) from August, 1999 to January, 2003. Centers were coordinated jointly by the US National Cancer Institute (NCI) and IARC. Cases are defined as patients between the ages of 20 and 79 years newly diagnosed with histologically confirmed RCC [ICD-O2; C64, International Classification of Disease for Oncology, Second Revision [12]]. Information on date and method of RCC diagnosis, tumor location, and stage and grade was abstracted from hospital records by trained medical staff. Controls were selected at each center among subjects admitted as in-patients or out-patients in the same hospital as the cases, with non-tobacco-related conditions and were frequency matched with cases by sex and age (+/− 3 years), and by study center. Patients with cancer or genitourinary disorders except for benign prostatic hyperplasia were also excluded from the controls. Although controls had to be cancer-free at the time of enrollment, previous history of cancer was not an exclusion criterion in either cases or controls. No single disease made up more than 20% of the diseases among selected controls from each center. Diagnoses of controls included digestive (20.3%), central nervous system (14.3%), eye and ear (16.9%), and musculoskeletal/connective tissue diseases (12.1%). Overall, 1097 cases and 1555 controls were interviewed, with response rates that ranged from 90 to 98.6% across study centers. Face to face interviews were conducted at each center. Collection of general demographic and risk factor information has been previously described [13], [14]. Blood samples were collected and stored at −80°C. Genomic DNA was extracted from whole blood buffy coat using standard phenol chloroform methods. In total, DNA was isolated from 987 cases and 1298 controls for genotyping. Informed consent was obtained from all participants, and the protocol was approved by the appropriate institutional review boards at each center.

### Genotyping

Genotyping was performed using two different methods:

1. TaqMan (Applied Biosysytems, Foster City, CA) assays were used to genotype genomic DNA for the SNP rs1800435. This SNP was chosen based on its functional relevance.
2. To comprehensively evaluate common variation across the ALAD gene region, GoldenGate (Illumina®, San Diego, CA) assays were used to analyze 18 additional SNPs (Table 1). SNP selection favored those with an expected minor allele frequency of >0.05 in Caucasians, those previously evaluated in the ALAD gene, and non-synonymous SNPs with potential functional relevance.

**Table 1:** *ALAD* tagging Single Nucleotide Polymorphisms (SNPs) evaluated in the Central Eastern European Renal Cancer Study.

Chromosome location	Location of Nucleotide Change	Amino Acid Change	dbSNP ID	Minor allele frequency in control population	Assay (Golden Gate or Taqman)
9q33	g.-22966C>T		rs14419	0.36	GoldenGate
	g.-19563T>A		rs818694	0.30	GoldenGate
9q32	g.IVS1+1425G>C		rs818688	0.37	GoldenGate
	g.IVS1+2615G>T		rs818687	0.18	GoldenGate
	g.IVS1−3185G>A		rs2792818	0.17	GoldenGate
	g.IVS2+299C>T		rs8177796	0.08	GoldenGate
	g.IVS3−196G>A		rs8177800	0.08	GoldenGate
	Ex4+13G>C	K68N	rs1800435	0.08	Taqman
	g.IVS4−139G>A		rs2761016	0.44	GoldenGate
	g.Ex6+17C>T	N147N	rs2228083	0.11	GoldenGate
	g.IVS11+66T>C		rs1805313	0.38	GoldenGate
	g.Ex12+533C>T		rs818708	0.44	GoldenGate
	g.Ex12+675G>A		rs818707	0.10	GoldenGate
	g.Ex12+100C>G		rs818705	0.18	GoldenGate
	g.Ex12+277C>T		rs818704	0.13	GoldenGate
	g.Ex12+352C>T		rs7042485	0.29	GoldenGate
	g.6440T>C		rs16933168	0.16	GoldenGate
	g.7758C>T		rs16936474	0.16	GoldenGate
	g.8429C>G		rs3750526	0.09	GoldenGate

Table 1 lists all SNPs genotyped using either the TaqMan® or GoldenGate method. SNPs were selected to provide high genomic coverage across *ALAD*. SNPs with minor allele frequencies of at least 5% and an r^2^ of ≥.80 within the genomic regions 20 kb 5′ of the start of transcription and 10 kb 3′ of the final exon (based on HapMap CEU data [15] were included. Nonsynonymous SNPs or those correlated with polymorphisms having potential functional significance were also included in this analysis. All genotyping was performed at the Core Genotyping Facility of the Division of Cancer Epidemiology and Genetics, National Cancer Institute (Frederick, MD). Detailed methods for all genotyping assays can be found at http:snp500cancer.nci.nioh.gov [16]. Genotyping was conducted by lab staff blinded to case/control status. Duplicate genotyping was performed for a randomly selected 5% of the total series for quality control purposes. The completion rate for all SNPs ranged from 98–100%. Concordance was >98% for all SNPs except rs1693474 [(7758 C>T) concordance = 95.51%]. The genotype frequencies among controls showed no deviation from the expected Hardy-Weinberg equilibrium proportions (p>0.05).

### Lead Exposure Assessment

Exposure was estimated using a thorough questionnaire-based exposure assessment strategy that has been previously described [11]. Briefly, face-to-face interviews were performed at each center. The questionnaire was administered by trained interviewers blinded to case-control status. Cases and controls were asked about their lifestyle habits, including such things as smoking and family medical history. This general questionnaire including a description of the tasks, machines used, working environment and time spent on each task was used for each job held at least one year. A second, specialized occupational questionnaire was administered in cases of employment in specific jobs or industries likely to entail exposure to lead. Details on the questionnaires have been previously reported [17]. For each job held, a team of chemists, industrial hygienists and occupational physicians evaluated lead exposure. Based on the general occupational questionnaire, the specialized questionnaires, and the assessor's own experience in industrial hygiene and knowledge about historical working conditions and tasks performed in the study areas, the frequency, intensity, and confidence of exposure were estimated [17]. Being ever exposed to lead was defined as someone who had held a job for more than 1 year at which they were ever exposed to lead dust or fumes. Frequency and intensity of lead exposure were not evaluated in this study.

### Statistical Analysis

For each SNP, we estimated the odds ratio (OR) and 95% confidence intervals (95% CI) using unconditional logistic regression. Unless otherwise stated; statistical analyses were performed using STATA version 10.0 (StataCorp, College Station, TX). Based on findings from previous studies, known or suspected risk factors for RCC (listed in Table 2) were included in the regression model individually to determine significance (p = <0.05). We then used forward selection to add significant variables to the model. All logistic regression models were adjusted for gender, age (categorical), and center (Czech Republic is a combination of 4 separate centers in Brno, Olomouc, Prague and Ceske). Smoking status, body mass index, self-reported hypertension, and family history of cancer did not alter the risk estimates by more than 10%; therefore these variables were not included in the final model. ORs were analyzed using the major homozygous allele as the referent group and separately comparing the heterozygous and homozygous minor alleles to subjects homozygous for the major allele as the referent. When the number of observations in the heterozygous or homozygous rare allele groups was less than 5% of the total genotypes among controls, the two categories were combined and compared to the referent group.

**Table 2:** Demographic characteristics of Subjects Genotyped in the Central Eastern European Renal Cancer Study.

CharacteristicTotal	CasesN (%)987	ControlsN (%)1298	ap-value-
Centerb,c			
Bucharest, RomaniaLodz, PolandMoscow, RussiaCzech Republic^b^	91 (9.2)81 (8.2)288 (29.2)527 (53.4)	132 (10.2)197 (15.2)368 (28.4)601 (46.3)	<0.0001
Sex			
MaleFemale	589 (59.7)398 (40.3)	838 (64.6)460 (35.4)	0.02
Age at Interview			
≤4445–5455–6465–74≥75	76 (7.7)253 (25.6)303 (30.7)313 (31.7)42 (4.3)	108 (8.3)333 (25.7)405 (31.2)397 (30.6)55 (4.2)	0.58
Smoking Statusc, d			
NeverEverMissing	454 (46.0)531 (53.8)2	528 (40.7)769 (59.2)1	0.01
Body Mass Index			
<2525–29≥30	186 (18.8)531 (53.8)270 (27.4)	321 (24.7)693 (53.4)284 (21.9)	<0.0001
Self-reported Hypertension			
NoYesMissing	539 (54.6)447 (45.3)1	800 (61.6)497 (38.3)1	0.001
Family history of Cancer^e^			
NoYes	654 (66)333 (34)	932 (72)366 (28)	0.004
Exposed to Lead^f^			
NeverEverMissing	675(68.4)71(7.2)241(24.4)	976(75.1)63(4.9)259(20.0)	0.001

a p-value unadjusted.

b Four centers: Brno, Olomuc, Prague, and Ceske.

c Totals do not equal 100% due to rounding.

d Not significant after adjustment for sex, age, and center.

e First degree relative with any cancer.

f Ever exposed to lead is defined as someone who has held a job for more than 1 year at which they were ever exposed to lead dust or fumes.

Haplotypes in the candidate block were analyzed using an R package Haplostats (Version 1.3.1) in (version 2.4.1), adjusting for gender, age, and center. The most common haplotype was used as the reference group and rare haplotypes (frequencies of <2%) were combined. We applied a haplotype-based sliding window approach with a fixed window size of 5 consecutive SNPs along the candidate gene. To test if specific haplotypes were associated with renal cancer risk, both a score and likelihood ratio test (LRT) were conducted. A global test that took into account all constructed haplotypes within a given haplotype block was conducted using a score test. SNP rs1800435 (Ex4 +13G>C (K68N)) was excluded from this analysis due to a high rate of missing data.

Heterogeneity of genotype frequencies between centers was evaluated by using the LRT. We found little evidence of heterogeneity across study centers. Moreover, no evidence of population stratification was apparent from a principal components analysis of a genome wide association study conducted in this population [18], and the likelihood of this is small among European populations [19]. To assess effect modification by risk factors of interest, we initially stratified the genotype analysis. Analysis to examine the joint effect of lead and genotype on RCC risk was then examined by adjusted logistic regression using a common referent group and an LRT for interaction (p = <0.05 significance).

## Results

Genotyping data was available on 987 (84.2%) cases and 1,248 (80.2%) of controls (Table 2). Subjects not genotyped were similar to those included in this study with respect to age, gender, and other known RCC risk factors (data not shown). The study population was of Caucasian descent, mostly male (60%) between 55–74 years of age (63%). Cases tended to be heavier and were more likely to report a history of hypertension and family history of cancer than controls. We first explored the association between lead exposure and RCC risk. After adjusting for age, gender, and center, the overall risk of RCC was increased among participants exposed to any lead (OR = 1.70, 95%CI = 1.21–2.38; p-value = 0.002) when compared to those with no reported lead exposure (data not shown). Using logistic regression across all included SNPs, we found little evidence of inter-country *ALAD* heterogeneity. In addition, we recently reported no evidence of lead exposure heterogeneity between countries (OR for exposure with a p-value = 0.12) [11].

### Individual SNP and Haplotype Analysis

When the main effects of individual SNPs were analyzed, those that carried the T allele at SNP rs8177796^CT/TT^ had a significantly higher risk of RCC compared to the common homozygote genotype; OR = 1.35, 95%CI = 1.05–1.73; p-value = 0.02 (Table S1). We did not observe any interaction with age, gender, reported hypertension, or smoking. No association was observed with the reported functionally rs1800435^GC/CC^ ^(K68N)^ variant. Based on our *a priori* hypothesis, we evaluated whether ALAD genotypes modified the risk of RCC associated with lead exposure (Table S1). Exposure to lead appeared to further increase RCC risk associated with the rs8177796^CT/TT^ variant when compared to the common genotype (OR = 1.67, 95%CI = 0.58–4.75, p-value = 0.34), but the estimates did not reach statistical significance. Compared to the common homozygote genotype, risk was elevated to OR = 1.15 and OR = 1.31 in individuals carrying the GA and AA genotype, respectively, who were not exposed to lead. Among those with lead exposure, the corresponding ORs were 0.59 and 0.29, respectively. However, none of the ORs reached statistical significance. No other tagging SNPs studied within the *ALAD* gene indicated a significant change in RCC risk when evaluated by lead exposure.

To further evaluate whether *ALAD* genotypes alter the relationship between lead and RCC, we modeled the joint effect of genotype and lead exposure (Table 3). Risk remained elevated among individuals not exposed to lead with the rs8177796 ^CT/TT^ (OR = 1.34, 95%CI = 1.00–1.80, p-value = 0.06) variant compared to the most common genotype referent. Exposure to lead further elevated the observed risk of RCC among participants having the rs8177796^CT/TT^ variant (OR = 2.52, 95%CI = 1.0–6.35, p = 0.05), but the interaction was not significant (p-value for interaction = 0.74). For participants with the wild-type G allele at the tagging SNP rs2761016, we observed an increase in RCC risk among participants exposed to lead (^GG^ genotype: OR = 2.68, 95%CI = 1.17–6.12; ^GA^ genotype: OR = 1.79, 95%CI = 1.06–3.04; ^AA^ genotype OR = 0.82, 95%CI = 0.29–2.35) with an interaction of borderline significance (p-value for interaction = 0.06). No significant alterations in risk were observed for the other tagging SNPs studied. Interestingly, after consideration of lead exposure in the joint model, the odds ratio was significantly elevated among subjects with the functionally relevant SNP rs1800435 among those with the GG genotype (OR = 1.83; 1.14–2.91, p = 0.01) but not among subjects with the GC/CC genotype (OR = 1.14; 0.35–3.69, p = 0.83); however the interaction was not significant (p-int = 0.43). After examination of correlation (r^2^) values between renal ALAD gene tag SNPs in Haploview, we observed that in our genotyped population SNPs rs16933168 and rs1693474 were highly correlated (r^2^ = 0.99). This r^2^ value was greater than that observed between tagging SNPs in HapMap at the time of SNP selection, which ranged from 80–90% at the time of selection (Figure S1).

**Table 3:** Odds ratio (OR) and 95% confidence intervals (CI) for joint ALAD tagging SNP genotypes, occupational lead exposure and renal cancer risk.

		*No Lead Exposure*			*Lead Exposure*			
SNP	Cases/Controls	aOR	95% CI	p-value^b^	aOR	95% CI	p-value^b^	cp-int
rs818687 *IVS1+2615G>T*								
GG	495/678	1.0	Referent		1.43	0.87, 2.36	0.16	
GT/TT	278/353	1.13	0.89, 1.45	0.31	2.05	1.06, 3.96	0.03	0.43
rs2792818 *IVS1+1425G>A*								
GG	527/698	1.0			1.59	0.91, 2.07	0.05	
GA/AA	247/337	1.0	0.78, 1.29	0.98	1.48	0.69, 3.17	0.31	0.65
rs8177796 *IVS2+299C>T*								
CC	624/872	1.0			1.51	0.97, 2.34	0.07	
CT/TT	153/161	1.34	1.00, 1.80	0.06	2.52	1.0, 6.35	0.05	0.74
rs8177800 *IVS3-196G>A*								
GG	647/865	1.0			1.41	0.92, 2.19	0.12	
GA/AA	130/167	0.86	0.63, 1.18	0.36	2.19	0.84, 5.72	0.11	0.17
rs1800435 *Ex4+13G>C (K68N)*								
GG	642/822	1			1.83	1.14, 2.91	0.01	
GC/CC	107/140	1.03	0.73, 1.46	0.87	1.14	0.35, 3.69	0.83	0.43
rs2761016 *IVS4-139G>A*								
GG	216/318	1.0			2.68	1.17, 6.12	0.01	
GA	403/523	1.15	0.88, 1.50	0.30	1.79	1.06, 3.04	0.05	
AA	158/191	1.31	0.94, 1.84	0.10	0.82	0.29, 2.35	0.68	0.06
rs2228083 (*Ex6+17C>T, N147N*)								
CC	612/815	1.0			1.44	0.92, 2.24	0.11	
CT/TT	165/217	1.03	0.77, 1.37	0.84	2.28	0.94, 5.51	0.07	0.46

a Odds ratios, 95% CI and.

b p-values were calculated from conventional logistic regression models adjusted for age, sex, and center.

c Tests for interaction for genotypes and lead exposure were calculated using a likelihood ratio test adjusting for age, sex, and region.

An unadjusted sliding window analysis of consecutive SNPs identified a 5-SNP region with a high level of signal, spanning the first 4 introns of the *ALAD* gene (Figure S1, global p-value = <0.01). When this region was evaluated in an adjusted model, several haplotypes were associated with an increased RCC risk (Table 4). The strongest association was observed with the G-C-T-G-G- haplotype (OR = 1.55; 95%CI: 1.16–2.06, p = <0.01). This association appears to be driven by the T allele at rs817796 (intron 2), which was significantly associated with increased RCC risk in the single SNP analysis (Table S1). We did not have statistical power to evaluate risk stratified by lead utilizing the haplotype model.

**Table 4:** ALAD Haplotype Association and Renal Cancer Risk.

Haplotype	Cases (%)	Controls (%)	OR^a^	95%CI	Adjustedp-value
*Region 1* (chr9:13284-5759)					
G-G-C-G-A	29.1	33.0	1		
G-G-C-G-G	23.0	21.6	1.21	0.97, 1.49	0.08
G-G-C-A-A	7.8	8.0	1.13	0.85, 1.49	0.38
G-C-C-G-A	7.2	6.8	1.23	0.88, 1.70	0.21
G-C-C-G-G	1.8	4.0	0.53	0.30, 0.93	0.03
G-C-T-G-G	8.1	5.9	1.55	1.16, 2.06	0.002
T-G-C-G-A	8.8	7.6	1.28	0.94, 1.74	0.11
T-G-C-G-G	10.8	10.6	1.15	0.89, 1.47	0.27
brare	3.5	2.5	1.50	0.95, 2.36	0.08

a Adjusted for age, sex, and center.

b Rare haplotypes (<2%) were combined into one category.

SNP's included in haplotype region 1: rs818687 (*IVS1+2615G>T*), rs2792818 (*IVS1+1425G>C*), rs8177796 (*IVS2+299C>T*), rs8177800 (*IVS3−196G>A*), rs2761016 (*IVS4−139A>G*).

## Discussion

In this study, overall lead exposure was associated with an increased risk of RCC, and this risk was modified by some ALAD genotypes. The increased risk associated with lead exposure was highest among subjects that had heterozygous or homozygous variants of the rs8177796 polymorphism and those with the common genotype at SNPs rs8177796 and rs2761016. We found a suggestion of decreased risk among participants with the rs2761016 homozygous variant who were exposed to lead. In addition to finding that occupational lead exposure increased RCC risk, we observed some *ALAD* variants alone altered cancer risk, independent of lead exposure. Confirmation of our findings will require replication in other large sufficiently powered studies with extensive lead exposure data.

If the risk of RCC is truly increased in individuals with *ALAD* polymorphisms, the question of mechanism depends on whether the association is dependent upon exogenous chemical exposures that disrupt the heme synthesis pathway or whether it occurs independently of exposure. The ALAD polymorphisms may encode an enzyme that is less active that the wild type, resulting in an accumulation of 5-aminolevulinic acid (ALA), a precursor thought to be genotoxic [[20], [21], reviewed in [22]]. However, ALAD enzymatic activity has been shown to not be significantly different [23] when comparing SNPs that result in changes in the translated sequence such as rs1800435 (K68N). It is possible that the increased risk of RCC in *ALAD* genotypic variants observed is due in part to exogenous chemicals that alter the heme synthesis pathway. Inhibition of ALAD enzymatic activity has been reported for multiple chemicals, including trichloroethylene, bromobenzene, styrene, and lead [24]. Polymorphic differences for enzyme inhibition have been most notably studied for lead. Individuals with the polymorphism at position 177 leading to a G→C transversion (rs1800435) results in three isozymes with different affinities for lead binding [8], [25]. The homozygous variant has a higher binding affinity for lead and has been associated with increased blood lead levels [8], [25], [26], [27]. The biological relevance of this alteration in lead binding is currently being debated in the literature. Studies have suggested that carriers of the G177C polymorphism are more susceptible to lead toxicity [7], [8]. Other studies suggest that the enhanced ability for the ALAD isozyme to bind lead actually confers a protective effect by sequestering circulating lead, slowing its accumulation in the bone marrow [28]. In this study, we observed a decrease in risk among those exposed to lead who carry the C allele at rs1800435^(K68N)^ compared to the wildtype G allele, however the number of cases did not provide a stable estimate.

The observation that individuals with the rs8177796 homozygous minor allele have an increased risk of RCC independently of lead exposure may be due to alterations in transcription. The SNP lies within an intronic region; however it is plausible that the polymorphism alters transcription of the wild-type *ALAD*, resulting in translation of a less active ALAD isozyme. It is also possible that the risk associated with the rs8177796 wild type variant does not represent a change in ALAD, but instead is in high linkage equilibrium with a biologically significant polymorphism that was captured by our tag SNP, but was not part of our examination. In contrast, we observed a decrease in RCC risk and significant interaction with lead exposure for the rs2761016 polymorphism. Similar to rs8177796, this intronic SNP may be altering ALAD activity, or could simply be tagging a region harboring an unidentified polymorphism. Additional genotyping to identify functional variants and *in vitro* analysis are needed to further explore the impact these intronic polymorphisms on ALAD activity.

The question of renal cancer risk associated with *ALAD* genotype has not been previously addressed in the literature. Recent work on ALAD polymorphsims and risk of brain tumors suggests an increased risk for meningioma among participants with the *ALAD* G177C homozygous genotype [29]. Schober et al. reported an increased risk of all cause, cardiovascular, and cancer mortality [30] associated with blood lead levels as low as 5–9 ug/dL [30]; however this study did not analyze the role of the ALAD genotype. We did not observe a significant change in RCC risk among participants with the G177C major allele overall or among lead exposed subjects. This variant was quite rare in this population and the number of overall cases exposed to lead was small, there-by under powering this gene-exposure interaction analysis. We chose to evaluate polymorphisms in *ALAD* based on the *a priori* biological and functional considerations, not by screening a large number of associations. Nonetheless, the possibility that our findings are due to chance cannot be ruled out. The results should be considered as hypothesis generating and require confirmation by replication in other studies.

A limitation of a hospital-based case-control study of occupational exposures is that the distribution of exposed participants might not be representative of the underlying healthy population. We attempted to address this issue by recruiting controls with a wide range of disease diagnoses. Apart from the neurological conditions, diseases reported among controls in our study are not known to be associated with lead exposure. It is thought that hospitalized patients may have different smoking patterns compared to the general population. A recent meta analysis reported the association between smoking and RCC risk is weakest among hospital-based studies [31]. We attempted to control for possible selection bias by excluding controls with smoking-related diseases, however the high number of smokers among our control population may indicate a bias in our study. Given multiple centers and countries were used in our study, the potential for population stratification exists; however we found no evidence of heterogeneity. It is possible that population stratification remains, but this is unlikely in European populations [19]. The lack of environmental measurements of lead exposure is an additional limitation of our study, which relied upon retrospective recall by study participants regarding their work history and other risk factors. However, since both cases and controls were hospitalized patients, any bias in recall would likely be non-differential with respect to exposure, attenuating the observed risk. Residual confounding by environmental lead exposure is also a limitation. Exposure misclassification is a concern in studies based on retrospective assessment, potentially causing us to underestimate risk if it is non-differential between cases and controls. Finally, the small number of cases exposed to lead and carrying any one *ALAD* variant restricts the statistical power of our analysis.

The strengths of this study are the high participation rates thus minimizing the potential for selection bias. The large sample size provided sufficient statistical power to evaluate small associations between genotype (with a prevalence of at least 10%) and risk. However, due to the low exposure prevalence (∼6%), our power to detect gene-exposure interactions was limited. Our use of job-specific questionnaire models to collect individual, detailed exposure information and local, expert-based exposure assessments to evaluate exposure histories is considered a superior approach for retrospective assessment of occupational exposures in community-based studies [32]. Although we had limited power for evaluating risk with respect to lead exposure and *ALAD* homozygous minor alleles (particularly those SNPs with suggested functional relevance), this study is one of the largest case-control studies of RCC and occupational lead exposure to date. In addition to the small percentage of RCC can be explained by familial syndromes including von Hippel-Lindau and hereditary papillary renal carcinoma roughly 50% of RCC incidence is thought to be associated with obesity, hypertension, and smoking [33]. The cause of the remaining half of incident cases remains unknown. Therefore, this study was designed to assess occupational and genetic factors in relation to RCC risk in a region with the highest incident rates worldwide [34]. To clarify the role of lead in the observed relationship between *ALAD* variants and risk of RCC, it will be important to conduct detailed, lead exposure assessments to evaluate how lead exposure; in combination with *ALAD* genetic variants could alter cancer risk in other study populations.

## Supporting Information

Figure S1 **Summary of sliding window results and linkage disequilibrium plot of ALAD genotyped region.** **Top**: Global and min p-values associated with each 5-SNP sliding window. **Bottom**: Linkage disequilibrium plot; red color intensity is based D′ and logarithm of the odds of linkage (LOD) scores. Each square contains an r^2^ value. (*) represents the placement of the ninth tagging SNP (rs1800435^K68N^), not included in the sliding window analysis due to a high rate of missing data.
(DOC)

Table S1 **Complete list of ALAD gene tagging SNPs and risk of renal cancer before and after stratification by lead exposure.**
(DOC)
