# Supplementary material for: Comprehensive Analysis of 5-Aminolevulinic Acid Dehydrogenase (ALAD) Variants and Renal Cell Carcinoma Risk among Individuals Exposed to Lead
Source: PLoS One. 2011 Jul 20;6(7):e20432. doi: 10.1371/journal.pone.0020432 (PMC3140467; doi:10.1371/journal.pone.0020432)
Supplement: Figure S1 — Summary of sliding window results and linkage disequilibrium plot of ALAD genotyped region. Top: Global and min p-values associated with each 5-SNP sliding window. Bottom: Linkage disequilibrium plot; red color intensity is based D′ and logarithm of the odds of linkage (LOD) scores. Each square contains an r2 value. (*) represents the placement of the ninth tagging SNP (rs1800435K68N), not included in the sliding window analysis due to a high rate of missing data. (DOC) [file pone.0020432.s001.doc]

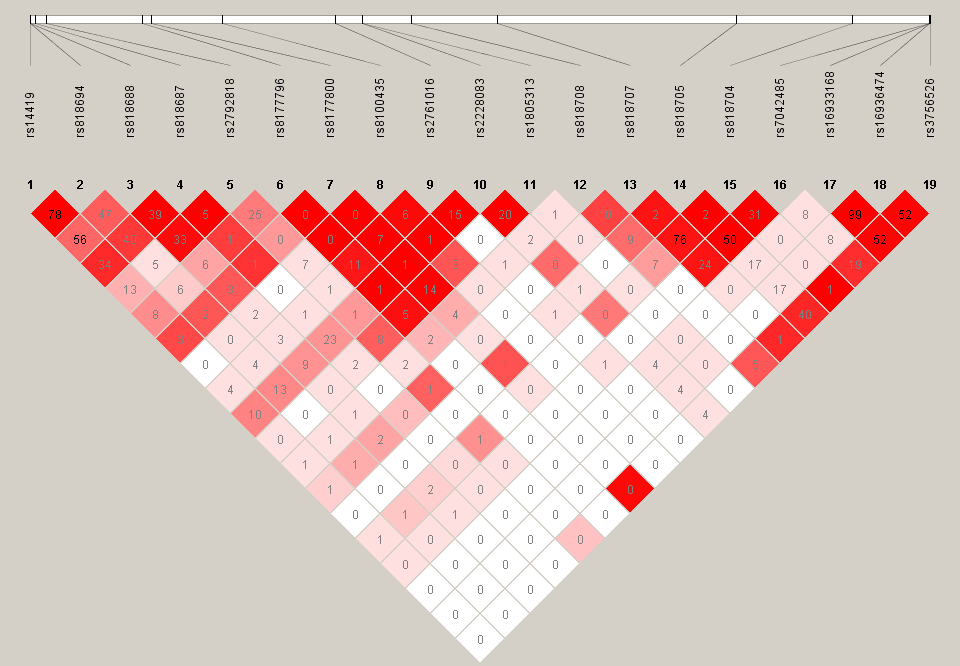


**Figure S1.** Summary of sliding window results and linkage disequilibrium plot of ALAD genotyped region.

Region 1

*****

1

2

3

4

5

6

7

8

15

12

11

13

14

19

16

17

18

10

*****

[Type a quote from the document or the summary of an interesting point. You can position the text box anywhere in the document. Use the Text Box Tools tab to change the formatting of the pull quote text box.]

[Type a quote from the document or the summary of an interesting point. You can position the text box anywhere in the document. Use the Text Box Tools tab to change the formatting of the pull quote text box.]

[Type a quote from the document or the summary of an interesting point. You can position the text box anywhere in the document. Use the Text Box Tools tab to change the formatting of the pull quote text box.]

[Type a quote from the document or the summary of an interesting point. You can position the text box anywhere in the document. Use the Text Box Tools tab to change the formatting of the pull quote text box.]
