# Supplementary material for: Comprehensive Analysis of 5-Aminolevulinic Acid Dehydrogenase (ALAD) Variants and Renal Cell Carcinoma Risk among Individuals Exposed to Lead
Source: PLoS One. 2011 Jul 20;6(7):e20432. doi: 10.1371/journal.pone.0020432 (PMC3140467; doi:10.1371/journal.pone.0020432)
Supplement: Table S1 — Complete list of ALAD gene tagging SNPs and risk of renal cancer before and after stratification by lead exposure. (DOC) [file pone.0020432.s002.doc]

| **Table S1.** Complete list of ALAD gene tagging SNPs and risk of renal cancer before and after stratification by lead exposure. | | | | | | | | | | | | | | | | | | | | | | | | | | | |  | | | |
| --- | --- | --- | --- | --- | --- | --- | --- | --- | --- | --- | --- | --- | --- | --- | --- | --- | --- | --- | --- | --- | --- | --- | --- | --- | --- | --- | --- | --- | --- | --- | --- |
|  | *All Subjects* | | | | | | | | | | *No Lead Exposure* | | | | | | | | | *Ever Occupationally exposed to Lead* | | | | | | | |  | | | |
| SNP | Cases/Controls | | | | ^a^OR | | | | 95% CI | | Cases/Controls | | | | ^a^OR | | 95% CI | | | Cases/Controls | | | ^a^OR | | 95% CI | | |  | | | |
| **rs14419 (-*22966C*>T)^b^** | | | | | | | | | | | | | | | | | | | | | | | | | | | |  | | | |
| CC | 313/417 | | | | 1 | | | |  | | | 209/312 | | 1 | | |  | | | 25/29 | | | 1 | |  | |  | | | | |
| CT | 344/501 | | | | 0.93 | | | | 0.76, 1.14 | | | 226/371 | | 0.91 | | | 0.72, 1.17 | | | 28/23 | | | 1.32 | | 0.59, 2.96 | |  | | | | |
| TT | 117/117 | | | | 1.34 | | | | 0.99, 1.80 | | | 74/79 | | 0.42 | | | 0.98, 2.05 | | | 6/4 | | | 1.61 | | 0.38, 6.84 | |  | | | | |
| p-trend |  | | | | 0.22 | | | |  | | |  | | 0.26 | | |  | | |  | | | 0.41 | |  | |  | | | | |
| **rs818694 (-*19563T*>*A*)^b^** | | | | | | | | | | | | | | | | | | | | | | | | | | | |  | | | |
| TT | 365/486 | | | 1 | | | |  | | | | 242/360 | | 1 | | | |  | | 26/35 | | | 1 | |  | |  | | | | |
| TA | 410/548 | | | 0.93 | | | | 0.76, 1.14 | | | | 211/348 | | 0.91 | | | | 0.72, 1.16 | | 28/18 | | | 2.08 | | 0.91, 4.76 | |  | | | | |
| AA | 86/80 | | | 1.43 | | | | 1.02, 2.00 | | | | 58/53 | | 1.68 | | | | 1.11, 2.54 | | 5/3 | | | 2.16 | | 0.43,10.77 | |  | | | | |
| p-trend |  | | | 0.27 | | | |  | | | |  | | 0.19 | | | |  | |  | | | 0.09 | |  | |  | | | | |
| **rs818688 (*IVS1+1425G>C*)** | | | | | | | | | | | | | | | | | | | | | | | | | | | |  | | | |
| GG | 299/408 | | | 1 | | | |  | | | | 198/313 | | 1 | | | |  | | 23/28 | | | 1 | |  | |  | | | | |
| GC | 358/498 | | | 0.98 | | | | 0.80, 1.21 | | | | 237/359 | | 1.04 | | | | 0.82, 1.33 | | 28/22 | | | 1.56 | | 0.68, 3.56 | |  | | | | |
| CC | 119/129 | | | 1.24 | | | | 0.93, 1.66 | | | | 77/90 | | 1.35 | | | | 0.94, 1.93 | | 7/6 | | | 1.51 | | 0.42, 5.43 | |  | | | | |
| p-trend |  | | | 0.28 | | | |  | | | |  | | 0.15 | | | |  | |  | | | 0.34 | |  | |  | | | | |
| **rs818687 (*IVS1+2615G>T*)** | | | | | | | | | | | | | | | | | | | | | | | | | | | |  | | | |
| GG | 495/678 | | | 1 | | | |  | | | | 327/509 | | 1 | | | |  | | 35/39 | | | 1 | |  | |  | | | | |
| GT/TT | 278/353 | | | 1.05 | | | | 0.86, 1.29 | | | | 183/249 | | 1.13 | | | | 0.89, 1.45 | | 24/17 | | | 1.5 | | 0.63, 3.56 | |  | | | | |
| p-value |  | | | 0.61 | | | |  | | | |  | | 0.31 | | | |  | |  | | | 0.25 | |  | |  | | | | |
| **rs2792818 (*IVS1-3185G>A*)** | | | | | | | | | | | | | | | | | | | | | | | | | | | |  | | | |
| GG | 527/698 | | | 1 | | | |  | | | | 344/519 | | 1 | | | |  | | 45/41 | | | 1 | |  | |  | | | | |
| GA/AA | 247/337 | | | 0.98 | | | | 0.80, 1.21 | | | | 165/243 | | 1.0 | | | | 0.78, 1.29 | | 14/15 | | | 0.95 | | 0.38, 2.34 | |  | | | | |
| p-value |  | | | 0.87 | | | |  | | | |  | | 0.99 | | | |  | |  | | | 0.91 | |  | |  | | | | |
| **rs8177796 (*IVS2+299C>T*)** | | | | | | | | | | | | | | | | | | | | | | | | | | | |  | | | |
| CC | 624/872 | | | 1 | | | |  | | | | 407/637 | | 1 | | | |  | | 47/48 | | | 1 | |  | |  | | | | |
| CT/TT | 153/161 | | | 1.35 | | | | 1.05, 1.73 | | | | 105/123 | | 1.34 | | | | 1.00, 1.80 | | 12/8 | | | 1.67 | | 0.58, 4.75 | |  | | | | |
| p-value |  | | | 0.02 | | | |  | | | |  | | 0.06 | | | |  | |  | | | 0.34 | |  | |  | | | | |
| **rs8177800 (*IVS3-196G>A*)** | | | | | | | | | | | | | | | | | | | | | | | | | | | |  | | | |
| GG | 647/865 | | 1 | | | |  | | | | | 428/623 | | 1 | | | |  | | 46/49 | | | 1 | |  | |  | | | | |
| GA/AA | 130/167 | | 1.0 | | | | 0.76, 1.29 | | | | | 84/136 | | 0.86 | | | | 0.63, 1.18 | | 13/7 | | | 1.65 | | 0.54, 5.03 | |  | | | | |
| p-value |  | | 0.98 | | | |  | | | | |  | | 0.37 | | | |  | |  | | | 0.38 | |  | |  | | | | |
| **rs1800435 (*Ex4+13G>C,* *K68N*)** | | | | | | | | | | | | | | | | | | | | | | | | | | | |  | | | |
| GG | 642/822 | | 1 | | | |  | | | | 415/604 | | | 1 | | | |  | | 47/36 | | | 1 | |  | | |  | | | |
| GC/CC | 107/140 | | 0.99 | | | | 0.75, 1.31 | | | | 65/91 | | | 1.03 | | | | 0.73, 1.46 | | 5/7 | | | 0.47 | | 0.12, 1.84 | | |  | | | |
| p-value |  | | 0.95 | | | |  | | | |  | | | 0.86 | | | |  | |  | | | 0.28 | |  | | |  | | | |
| **rs2761016 (*IVS4-139G>A*)** | | | | | | | | | | | | | | | | | | | | | | | | | | | |  | | | |
| GG | 216/318 | | 1 | | | |  | | | | | 142/240 | | 1 | | | |  | | 17/10 | | | 1 | |  | |  | | | | |
| GA | 403/523 | | 1.14 | | | | 0.92, 1.42 | | | | | 257/377 | | 1.15 | | | | 0.88, 1.5 | | 36/35 | | | 0.59 | | 0.22, 1.54 | |  | | | | |
| AA | 158/191 | | 1.22 | | | | 0.93, 1.61 | | | | | 113/142 | | 1.31 | | | | 0.94, 1.84 | | 6/11 | | | 0.29 | | 0.07, 1.06 | |  | | | | |
| p-trend |  | | 0.14 | | | |  | | | | |  | | 0.18 | | | |  | |  | | | 0.06 | |  | |  | | | | |
| **rs2228083 (*Ex6+17C>T, N147N*)** | | | | | | | | | | | | | | | | | | | | | | | | | |  | | |  |  |  |
| CC | 612/815 | | 1 | | | |  | | | | | | 401/601 | 1 | | | |  | | 45/47 | | | 1 | |  | |  | | | | |
| CT/TT | 165/217 | | 0.98 | | | | 0.77, 1.24 | | | | | | 111/158 | 1.03 | | | | 0.77, 1.37 | | 14/9 | | | 1.25 | | 0.44, 3.55 | |  | | | | |
| p-value |  | | 0.85 | | | |  | | | | | |  | 0.82 | | | |  | |  | | | 0.67 | |  | |  | | | | |
|  |  | |  | | | |  | | | | | |  |  | | | |  | |  | | |  | |  | |  | | | | |
| *Table Continues* | | | | | | | | | | | | | | | | | | | | | | | | | | | |  | | | |
|  | *All Subjects* | | | | | | | | | | | | *No Lead Exposure* | | | | | | | | *Ever Occupationally exposed to Lead* | | | | | |  | | | | |
| SNP | Cases/ controls | | | | | ^a^OR | | | | 95% CI | | | Cases/Controls | | | ^a^OR | | | 95% CI | | Cases/Controls | ^a^OR | | 95% CI | | |  | | | | |
| **rs1805313 (*IVS11+66T>C*)** | | | | | | | | | | | | | | | | | | | | | | | | | | | |  | | | |
| TT | 312/384 | | | | | 1 | | | |  | | | 200/277 | | | 1 | | |  | | 23/24 | 1 | |  | | |  | | | | |
| TC | 344/501 | | | | | 0.84 | | | | 0.68, 1.03 | | | 236/362 | | | 0.9 | | | 0.70, 1.15 | | 22/28 | 0.69 | | 0.29, 1.67 | | |  | | | | |
| CC | 120/143 | | | | | 1.01 | | | | 0.76, 1.35 | | | 75/116 | | | 0.92 | | | 0.65, 1.30 | | 14/4 | 3.13 | | 0.87,11.27 | | |  | | | | |
| p-trend |  | | | | | 0.60 | | | |  | | |  | | | 0.49 | | |  | |  | 0.20 | |  | | |  | | | | |
| **rs818708 (*Ex12+533C>T*)** | | | | | | | | | | | | | | | | | | | | | | | | | | | |  | | | |
| CC | 248/327 | | | | | 1 | | | |  | | | 173/227 | | | 1 | | |  | | 15/20 | 1 | |  | | |  | | | | |
| CT | 381/496 | | | | | 1.04 | | | | 0.84, 1.29 | | | 240/367 | | | 0.89 | | | 0.68, 1.15 | | 28/30 | 1.24 | | 0.51, 3.00 | | |  | | | | |
| TT | 148/208 | | | | | 0.96 | | | | 0.73, 1.26 | | | 99/164 | | | 0.82 | | | 0.60, 1.14 | | 16/6 | 3.57 | | 1.10,11.58 | | |  | | | | |
| p-trend |  | | | | | 0.84 | | | |  | | |  | | | 0.22 | | |  | |  | 0.05 | |  | | |  | | | | |
| **rs818707 (*Ex12+675G>A*)** | | | | | | | | | | | | | | | | | | | | | | | | | | | |  | | | |
| GG | 634/830 | | | | | 1 | | | |  | | | 414/599 | | | 1 | | |  | | 47/47 | 1 | |  | | |  | | | | |
| GA/AA | 143/203 | | | | | 0.9 | | | | 0.70, 1.15 | | | 98/161 | | | 0.88 | | | 0.66, 1.18 | | 10/8 | 1.47 | | 0.51, 4.22 | | |  | | | | |
| p-value |  | | | | | 0.42 | | | |  | | |  | | | 0.39 | | |  | |  | 0.47 | |  | | |  | | | | |
| **rs818705 (*Ex12+100C>G*)** | | | | | | | | | | | | | | | | | | | | | | | | | | | |  | | | |
| CC | 518/690 | | | | | 1 | | | |  | | | 331/514 | | | 1 | | |  | | 41/34 | 1 | |  | | |  | | | | |
| CG/GG | 257/341 | | | | | 1.01 | | | | 0.83, 1.24 | | | 180/244 | | | 1.15 | | | 0.90, 1.46 | | 18/22 | 0.77 | | 0.34, 1.78 | | |  | | | | |
| p-value |  | | | | | 0.90 | | | |  | | |  | | | 0.27 | | |  | |  | 0.55 | |  | | |  | | | | |
| **rs818704 (*Ex12+277C>T*)** | | | | | | | | | | | | | | | | | | | | | | | | | | | |  | | | |
| CC | 613/790 | | | | | 1 | | | |  | | | 398/568 | | | 1 | | |  | | 48/45 | 1 | |  | | |  | | | | |
| CT/TT | 164/243 | | | | | 0.85 | | | | 0.68, 1.07 | | | 114/193 | | | 0.82 | | | 0.63, 1.08 | | 11/11 | 1.08 | | 0.39, 2.96 | | |  | | | | |
| p-value |  | | | | | 0.17 | | | |  | | |  | | | 0.17 | | |  | |  | 0.88 | |  | | |  | | | | |
| **rs7042485 (*Ex12+352T>C*)** | | | | | | | | | | | | | | | | | | | | | | | | | | | |  | | | |
| TT | 380/490 | | | | | 1 | | | |  | | | 239/355 | | | 1 | | |  | | 30/27 | 1 | |  | | |  | | | | |
| TC | 340/453 | | | | | 0.95 | | | | 0.78, 1.16 | | | 229/338 | | | 0.99 | | | 0.78, 1.26 | | 25/23 | 0.99 | | 0.45, 2.19 | | |  | | | | |
| CC | 57/90 | | | | | 0.81 | | | | 0.56, 1.16 | | | 44/67 | | | 0.92 | | | 0.61, 1.40 | | 4/6 | 0.56 | | 0.13, 2.32 | | |  | | | | |
| p-trend |  | | | | | 0.28 | | | |  | | |  | | | 0.76 | | |  | |  | 0.57 | |  | | |  | | | | |
| **rs16933168 (*6440T>C*)^b^** | | | | | | | | | | | | | | | | | | | | | | | | | | | |  | | | |
| TT | 539/721 | | | | | 1 | | | |  | | | 346/530 | | | 1 | | |  | | 40/42 | 1 | |  | | |  | | | | |
| TC/CC | 238/313 | | | | | 1.0 | | | | 0.81, 1.18 | | | 166/231 | | | 1.04 | | | 0.81, 1.34 | | 18/14 | 1.60 | | 0.66, 3.91 | | |  | | | | |
| p-value |  | | | | | 0.97 | | | |  | | |  | | | 0.43 | | |  | |  | 0.30 | |  | | |  | | | | |
| **rs16936474 (*7758C>T*)^b^** | | | | | | | | | | | | | | | | | | | | | | | | | | | |  | | | |
| CC | 539/718 | | | | | 1 | | | |  | | | 346/527 | | | 1 | | |  | | 40/42 | 1 | |  | | |  | | | | |
| CT/TT | 235/311 | | | | | 0.98 | | | | 0.80, 1.21 | | | 165/230 | | | 1.03 | | | 0.80, 1.32 | | 19/13 | 1.81 | | 0.72, 4.53 | | |  | | | | |
| p-value |  | | | | | 0.89 | | | |  | | |  | | | 0.82 | | |  | |  | 0.30 | |  | | |  | | | | |
| **rs3756526 (*8429C>G*)^b^** | | | | | | | | | | | | | | | | | | | | | | | | | | | |  | | | |
| CC | 624/847 | | | | | 1 | | | |  | | | 401/624 | | | 1 | | |  | | 49/46 | 1 | |  | | |  | | | | |
| CG/GG | 152/183 | | | | | 1.11 | | | | 0.88, 1.44 | | | 110/133 | | | 1.26 | | | 0.94, 1.68 | | 10/10 | 1.01 | | 0.36, 2.78 | | |  | | | | |
| p-value |  | | | | | 0.35 | | | |  | | |  | | | 0.13 | | |  | |  | 0.99 | |  | | |  | | | | |
| ^a^Adjusted for age, sex, and center.  ^b^Chromosomal location relative to *ALAD* ATG site.  When the number of observations in the heterozygous or homozygous rare allele groups was less than 5% of the total genotypes among controls, the two categories were combined and compared to the referent group. | | | | | | | | | | | | | | | | | | | | | | | | | | | |  | | | |
|  | |  | | | | | | | | | | | | | | | | | | | | | | | | | |  | | | |
